# Supplementary material for: Oxyntomodulin regulates resetting of the liver circadian clock by food
Source: eLife. 2015 Mar 30;4:e06253. doi: 10.7554/eLife.06253 (PMC4426666; doi:10.7554/eLife.06253)
Supplement: Figure 6—source data 1. — DOI: http://dx.doi.org/10.7554/eLife.06253.012 [file elife06253s002.doc]

**Figure 6 – source data 1.** PCR primer sequences

|  | **Forward primer** | **Reverse primer** |
| --- | --- | --- |
| **Per1** | AGTTCCTGACCAAGCCTCGTTAG | CCTGCCCTCTGCTTGTCATC |
| **Per2** | GCCAAGTTGTGGAGTTCCT | CTTGCACCTTGACCAGGTAGG |
| **Dbp** | AATGACCTTTGAACCTGATCCCGCT | GCTCCAGTACTTCTCATCCTTCTGT |
| **Bmal1** | CCTAATTCTCAGGGCAGCAGAT | TCCAGTCTTGGCATCAATGAGT |
|  |  |  |
| **FOXO1** | TGGTCAAGAGCGTGCCTACT | CCCGCTCTTGCCACCCTCTG |
| **Pdk4** | AGGGAGGTCGAGCTGTTCTC | GGAGTGTTCACTAAGCGGTCA |
| **Pklr** | CGTTTGTGCCACACAGATGCT | CATTGGCCACATCGCTTGTCT |
| **Pepck1** | CACCATCACCTCCTGGAAGA | GGGTGCAGAATCTCGAGTTG |
| **Slc2a2** | TCAGAAGACAAGATCACCGGA | GCTGGTGTGACTGTAAGTGGG |
| **Slc16a7** | CACCATCACCTCCTGGAAGA | GGGTGCAGAATCTCGAGTTG |

Glp1r^-/-^ Genotyping

| **oIMR0013/14** | CTTGGGTGGAGAGGCTATTC | AGGTGAGATGACAGGAGATC |
| --- | --- | --- |
| **GLP1R F2/R2** | TACACAATGGGGAGCCCCTA | AAGTCATGGGATGTGTCTGGA |
